# Immunohistochemical Expression Pattern of Theragnostic Targets SSTR2 and PSMA in Endolymphatic Sac Tumors: A Single Institution Case Series

**DOI:** 10.1007/s12105-022-01456-3

**Published:** 2022-05-11

**Authors:** Muriel D. Brada, Elisabeth J. Rushing, David Bächinger, Loris Zoller, Irene A. Burger, Martin W. Hüllner, Holger Moch, Alexander Huber, Andreas H. Eckhard, Niels J. Rupp

**Affiliations:** 1 Department of Pathology and Molecular Pathology, University Hospital Zurich, Schmelzbergstrasse 12, 8091 Zurich, Switzerland; 2 Department of Neuropathology, University Hospital Zurich, Zurich, Switzerland; 3 Department of Otorhinolaryngology, Head and Neck Surgery, University Hospital Zurich, Zurich, Switzerland; 4 Department of Nuclear Medicine, Baden Cantonal Hospital, Baden, Switzerland; 5 Department of Nuclear Medicine, University Hospital Zurich, Zurich, Switzerland; 6 Faculty of Medicine, University of Zurich, Zurich, Switzerland

**Keywords:** Endolymphatic sac, von Hippel-Lindau syndrome, Early detection of cancer, Ear neoplasms, Endothelial cells, Positron emission tomography computed tomography

## Abstract

**Background:**

Endolymphatic sac tumors are rare neoplasia characterized by slow growth. However, their clinical impact should not be underestimated, considering their potential for local aggressive behavior and strong association with von Hippel–Lindau syndrome. Therefore, early detection with emerging theragnostic examinations such as ^68^Ga-DOTATATE-PET/CT might improve patient management and reduce morbidity.

**Methods:**

We report the clinicopathological features of seven endolymphatic sac tumors. In this cohort, we performed immunohistochemical analysis of somatostatin receptor 2A (SSTR2A) and prostate specific membrane antigen (PSMA) protein expression patterns; two targets providing rationale for novel imaging modalities such as PSMA- or SSTR-targeted PET.

**Results:**

The tumor cells of all cases were negative for prostate specific membrane antigen and somatostatin receptor 2A, however immunolabeling was consistently detected in intratumoral endothelial cells of endolymphatic sac tumors for PSMA (7/7 cases, 100%), and for SSTR2A (5/7 cases, 71%).

**Conclusions:**

Our results show a high rate of PSMA and SSTR2A expression in the tumor vasculature of endolymphatic sac tumors. PSMA and SSTR2A can be targeted with appropriate radioligands for diagnostic and therapeutic purposes. This finding provides a rationale for prospective clinical studies to test this approach as a sensitive screening tool for patients with suspected endolymphatic sac tumors including an improved management of von Hippel–Lindau syndrome.

## Introduction

Endolymphatic sac tumors (ELSTs) are low-grade malignant epithelial tumors of neuroectodermal origin arising in the endolymphatic sac of the inner ear, located in the temporal bone and the dura of the posterior cranial fossa. The endolymphatic sac has been associated with several functions in the inner ear, such as regulation of the endolymph ionic content and volume, as well as immunologic functions [1–3].

ELSTs are extremely rare and growing slowly, presenting with unspecific symptoms, including tinnitus, hearing loss, vertigo and facial palsy [4, 5]. ELSTs occasionally show invasive and locally destructive growth, infiltrating the middle ear, middle and posterior cranial fossae and the cerebellopontine angle, with exceptional cases giving rise to metastatic disease [6]. Histologically, they are typically small, well-vascularized neoplasms with variable papillary, glandular or cystic morphology with a single layer of neoplastic epithelial cells [7]. Age at diagnosis is highly variable and surgical excision represents the treatment of choice, with the possibility of radiotherapy in advanced cases. At least a third of ELSTs are detected in patients with VHL syndrome, with up to 30% occurring bilaterally. In this setting, ELSTs are usually detected at a younger age and often as the first disease manifestation [8].

Von Hippel–Lindau disease is an autosomal dominantly inherited tumor predisposition syndrome with germline mutations in a tumor suppressor gene on chromosome 3 (*VHL* gene 3p25.3) [9]. In addition to ELSTs, VHL patients typically develop multiple hemangioblastomas of the central nervous system, clear cell renal cell carcinomas (ccRCC), pheochromocytomas, extra-adrenal paragangliomas, as well as pancreatic adenomas and neuroendocrine tumors [10]. Inactivation of the VHL tumor suppressor gene due to genetic alterations of wild type alleles has been identified in VHL patients as well as in sporadic ELSTs [11, 12]. This finding was recently confirmed in a larger cohort. Of note, in a minority of cases without *VHL*-alterations, Schweizer et al. found *TERT* promotor mutations [13]. Interestingly, these findings are similar to clear cell renal cell carcinoma, which may also harbor inactivating *VHL* mutations in sporadic cases [14].

Bi-allelic inactivation of the *VHL* gene leads to constitutive activation of the hypoxia-inducible factors (HIF-1 and HIF-2), promoting neovascularization through downstream targets such as vascular endothelial growth factor (VEGF), which is strongly expressed in the tumor cells of ELSTs [15, 16].

Immunohistochemical studies have consistently reported that ELSTs are immunoreactive for specific cytokeratins (CK7, CK8, CK18), as well as epithelial membrane antigen (EMA), vimentin, glial fibrillary acidic protein (GFAP), S100, paired-box-proteins 8 and 2 (PAX-8/ PAX-2) and carbonic anhydrase IX (CAIX). In contrast, thyroid transcription factor 1 (TTF-1), anti-renal cell carcinoma (RCC), CD10, CK20, as well as synaptophysin, chromogranin A, thyroglobulin and transthyretin are negative [15, 17, 18]. Reported proliferation index is very low, ranging between 1 and 3%; however, cases showing progression and invasive behavior suggest the existence of possible clones with higher proliferative potential [15].

The early detection of limited disease remains crucial, with complete surgical excision representing the most successful therapy to limit morbidity and mortality [5]. Next to conventional computed tomography (CT) and magnetic resonance imaging (MRI), gallium-68 (^68^Ga)-DOTATATE positron emission tomography (PET)/CT technique has emerged as a potential screening tool for ELSTs, with the original aim of detecting VHL-associated tumors such as hemangioblastomas, pancreatic neuroendocrine tumors, as well as paragangliomas [19]. Initially, somatostatin receptor (SSTR) expression was suspected to reside on the cell-surface of tumor cells; however, localization of somatostatin receptor type 2A (SSTR2A) expression has only recently been reported in the tumor vasculature with corresponding low-level radiotracer uptake in the tumor area in a single case [20].

Furthermore, SSTR2A expression has gained importance as a possible prognostic biomarker as well as potential diagnostic and therapeutic (theragnostic) target in tumor entities such as neuroendocrine tumors or nasopharyngeal carcinoma [21–24]. In this so-called theragnostic approach, structures on tumor cells and/or in tumor-associated neovasculature are targeted for diagnostic and therapeutic purposes. This concept was first applied to thyroid diseases, where it is part of standard therapeutic practice [25]. Novel radioligands are currently becoming an integral part of the diagnosis and therapy of prostate adenocarcinoma and neuroendocrine tumors. Considering the emerging applications of this technique, the aim of our study is to further characterize the immunohistochemical expression patterns of SSTR2A in ELSTs. Due to the established use of PSMA-PET in the staging and follow up of prostate cancer patients, and recognition of PSMA expression in a variety of other tumor entities, PSMA staining was also evaluated as a potential theragnostic target.

## Materials and Methods

The study cohort consists of seven cases of ELSTs, all diagnosed between 1993 and 2018 at the Institute of Pathology and Molecular Pathology, in collaboration with the Institute of Neuropathology, University Hospital Zurich. All cases have been comprehensively reviewed by experienced head and neck pathologists (M.D.B., N.J.R.) and a neuropathologist (E.J.R.). Histopathological evaluation of hematoxylin and eosin (HE)-stained slides confirmed the initial diagnosis, with a representative tumor tissue block chosen for further investigations. Immunohistochemical staining of tumor was performed using commercially available antibodies on an automated staining system (Ventana Benchmark) with the following antibodies: pancytokeratin AE1/AE3 (monoclonal, 1:50, DAKO A/S); PAX8 (monoclonal, 1:100, Abcam Limited), somatostatin receptor 2A (SSTR2A) (polyclonal, 1:75, Zytomed Systems), Ki-67 (monoclonal, 30–9, prediluted) and prostate-specific membrane antigen (PSMA) (monoclonal, 1:25, DAKO A/S). Immunostaining for cytokeratin and PAX8 was assessed as either present or absent, while for SSTR2A and PSMA, staining intensity was scored as either negative (0), weak (1+), moderate (2+) or strong (3+). External controls for SSTR2A and PSMA included pancreatic and prostatic tissue.

## Results

### Clinical Data and Demographics

Table 1 contains clinical presentation, tumor size and location, as well as treatment approach of our cohort. The gender distribution (male:female ratio = 1.3:1) for the seven patients diagnosed with endolymphatic sac tumor was almost equal. Median age at diagnosis was 26 years and clinical information identified six patients with underlying von Hippel–Lindau (VHL) syndrome (6/7 cases, 86%). Available follow-up data of 6 patients with ELST, ranging from 12 to 130 months, showed disease recurrence in 2 patients (2/6, 33.3%), while one patient was lost to follow up.

**Table 1:** Cohort clinicopathological data: case nr. 1–7 endolymphatic sac tumor

Case number	Age at diagnosis	Gender	Tumor size (mm)	Tumor location	Morphology	Clinical presentation	Therapy
1	44	Male	10	Right cerebellopontine angle/posterior cranial fossa	Glandular-cystic	Hearing loss, tinnitus	Tumor resection by subtotal petrosectomy with resection of otic capsule
2	18	Female	12	Left vestibular aqueduct	Papillary/cystic	Vertigo, hearing loss	Tumor resection by subtotal petrosectomy without resection of otic capsule
3	23	Male	15	Left vestibular aqueduct	Papillary/cystic	Hearing loss, tinnitus	Translabyrinthine tumor resection
4	21	Female	23	Right posterior cranial fossa with labyrinthic infiltration	Papillary/cystic	No audiovestibular symptoms	Tumor resection by infratemporal fossa approach (Fisch type A) and subtotal petrosectomy
5	43	Male	13	Right posterior cranial fossa	Papillary/cystic	Deafness, vertigo	Tumor resection by subtotal petrosectomy with resection of otic capsule
6	14	Female	5	Right vestibular aqueduct	Papillary/cystic	Tinnitus	Transmastoid retrolabyrinthine tumor resection
7	20	Male	10	Left vestibular aqueduct and posterior semicircular canal	Papillary/cystic	No audiovestibular symptoms	Transmastoid tumor resection with partial resection of posterior semicircular canal

### Morphological Description

**Fig. 1:**
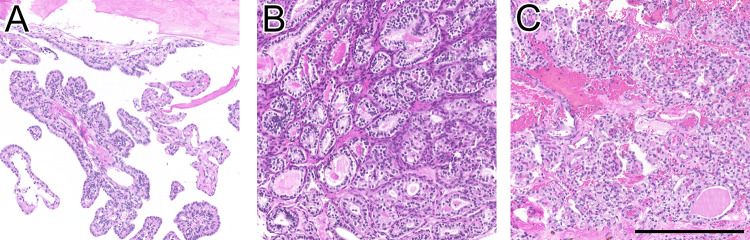
H&E-stained sections depicting the growth patterns of three representative ELSTs: **A** papillary; **B** glandular-cystic and **C** mixed papillary and glandular-cystic growth. Scale bar: 250 μm

The histomorphological spectrum of ELSTs included both papillary and glandular-cystic growth patterns, with admixed patterns in most cases (5/7 cases, 71.3%). Representative growth patterns are illustrated in Fig. 1. Interestingly, in one case, the tumor initially showed papillary features, while one year later at relapse, cystic growth pattern dominated.

### Immunohistochemical Expression Characteristics

All cases showed diffuse, robust staining for cytokeratin (AE1/AE3) with positivity for PAX8 in all ELSTs.

**Fig. 2:**
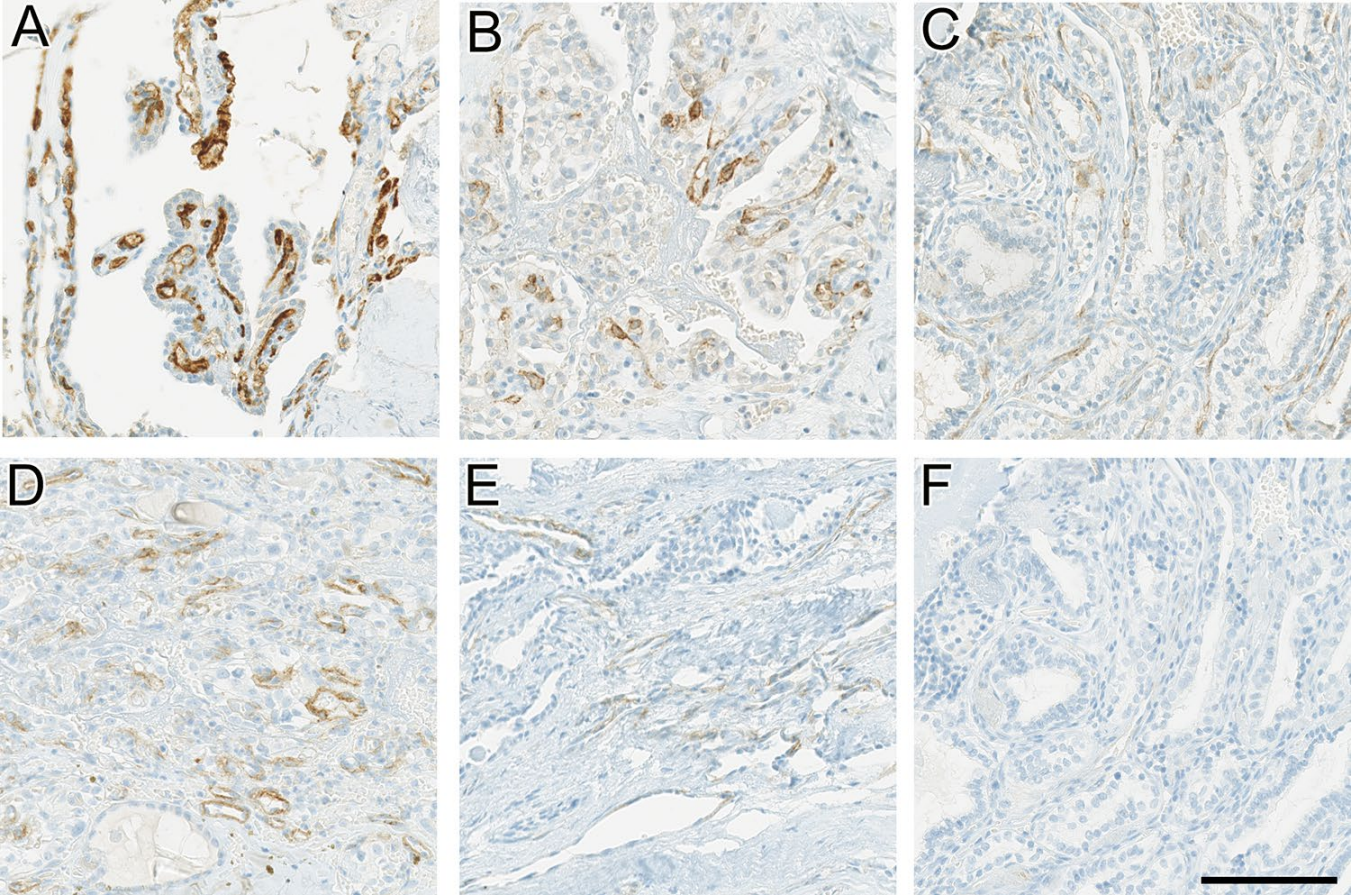
Immunohistochemical expression patterns of PSMA and SSTR2 in our cohort of ELSTs, displaying the various staining intensities in the tumor vasculature, while epithelial tumor cells lining papillae and glandular structures remain negative. For each staining intensity, one representative case is shown: **A** strong (3+) PSMA expression in endothelial cells. **B** and **C** moderate (2+) and weak (1+) PSMA expression, respectively. **D** moderate (2+) SSTR2A expression in endothelial cells of the tumor vasculature. **E** and **F** weak (1+) and negative (0) SSTR2A expression. Scale bar: 100 μm

Immunostainings for PSMA and SSTR2A were negative in the constituent tumor cells in all cases of ELST. However, tumor-associated vessels immunolabeled with PSMA in all cases, including four cases (4/7, 66.7%) with moderate (2+) to strong (3+) intensity, and three cases (3/7, 33.3%) with weak (1+) immunostaining. In five cases (5/7, 71.4%), SSTR2A immunostaining was weaker than PSMA staining, but clearly detectable: Staining intensity was moderate (2+) in a single case (1/7, 14.3%), and weak (1+) in four cases (4/7, 57.1%). Figure 2 illustrates representative immunohistochemical expression patterns for PSMA and SSTR2A. No expression of SSTR2A or PSMA was noted in adjacent normal tissue. Similar to previous reports, the Ki-67 proliferation rate was very low, not exceeding 1%. Table 2 summarizes the staining results of the seven ELSTs.

**Table 2:** Cohort immunohistochemical results (staining intensity scores)

Case number	PAX8	SSTR2A/PSMA tumor cells	SSTR2A vessels	PSMA vessels	MIB-1 (Ki-67)
1	1	0	0	1	< 1%
2	1	0	1	3	< 1%
3	1	0	1	1	< 1%
4	1	0	1	3	< 1%
5	1	0	2	2	< 1%
6	1	0	0	1	< 1%
7	1	0	1	3	< 1%

PAX8 (0 = negative, 1 = positive), SSTR2A/PSMA (0 = negative, 1 = weak, 2 = moderate, 3 = strong)

## Discussion

Endolymphatic sac tumors are part of the spectrum of mass lesions involving the inner ear. Their rarity as well as their characteristic slow growth accompanied by non-specific symptoms render the diagnosis challenging [5]. Conventional imaging techniques are limited in differentiating ELST from jugulotympanic paraganglioma, middle ear adenoma, meningioma, and choroid plexus papilloma [26]. Therefore, finding alternate screening modalities is of clinical interest and relevance.

To our knowledge, this represents the first study evaluating the expression of two commonly used radioligand targets, SSTR2A and PSMA, in a series of ELSTs. In more than 70% of our cases, we detected weak to moderate immunohistochemical expression of SSTR2A in the tumor vasculature of ELSTs, while the tumor cells remained negative. This staining pattern is in concordance with a recent case report of an ELST, in which the staining pattern was proposed as a histopathological correlate to weak but distinct tracer uptake in ^68^Ga-DOTATATE-PET/CT [20]. To date, two case reports have documented mild but distinct radiotracer uptake on ^68^Ga-DOTATATE-PET/CT of ELST (SUVmax 10.9 and 6.29) [19, 20]. The level of uptake was considerably lower than that reported for head and neck paragangliomas, an important differential diagnosis, indicating applicability as a possible clinical screening tool [27]. However, to the best of our knowledge, no prospective data have yet been reported on the correlation of endothelial SSTR2 expression in ELSTs and corresponding SSTR-targeting PET examinations. Paragangliomas and meningiomas strongly express SSTR2 on tumor cells, providing a rationale for the binding and accumulation of SSTR targeting agents such as DOTATATE [28, 29]. Middle ear adenoma, currently renamed as middle ear neuroendocrine tumor (MeNET) [30], usually show neuroendocrine differentiation and SSTR expression is indicated by positive octreotide scan [31]. Furthermore, high uptake on ^68^Ga-DOTATOC PET/CT followed by effective peptide receptor radionuclide therapy (PRRT) was reported [32]. Histologically, metastasis from renal, thyroid and prostate cancer might mimic ELST and need to be excluded.

Prostate-specific membrane antigen (PSMA)-PET has evolved as an important adjunct to conventional imaging techniques in the management of patients with prostate cancer [33]. Notably, immunohistochemical tumor expression patterns correlate with ^68^Ga-PSMA-11 accumulation in the staging and restaging setting of prostate cancer [34, 35].

Next to prostate cancer cells, PSMA targeted tracer uptake can be observed in normal tissue, with the strongest uptake in the kidneys and salivary glands [36]. However, in salivary glands evidence exists for a distinct PSMA-unrelated uptake mechanism [37, 38]. Additionally, PSMA targeted tracer uptake has been described in multiple malignancies from different organ systems, most likely attributable to PSMA expression in the endothelial cells of the tumor neovasculature. This observation has been consistently documented in clear cell renal cell carcinoma, and somewhat less consistently in breast cancer and non-small cell lung cancer [39]. Among the histologic mimics of ELSTs, thyroid and renal cancer showed variable PSMA expression in tumoral microvessels [39]. Here, we present first evidence for recurrent PSMA expression in the tumor neovasculature of ELSTs, comparable to the tumor entities described above. Even though staining intensity is variable, almost 70% of our cases show strong or clearly visible PSMA expression in tumor endothelial cells.

In summary, SSTR2, as well as PSMA are nonspecific markers found in many tumor entities with variable expression patterns. In the literature concordant data exist with our own experience of variable vascular PSMA and SSTR2 expression in clear cell renal cell carcinoma (unpublished data), as the morphologically closest differential diagnosis [39–41]. With our results, we added ELST to the list, although with a recurrent vascular expression pattern in a high percentage of cases for both markers. PSMA and SSTR2 do not aid in histopathologic diagnosis of this entity, which leans on morphology and other more specific immunohistochemical markers. However, our findings highlight the potential utility of SSTR- and/or PSMA-targeting PET examinations in the detection of ELSTs.

Limitations to our retrospective study include the lack of a preoperative PET/CT or PET/MR scan to demonstrate a relationship with possible radiotracer uptake. Further, our cohort is relatively small, yet representative, considering the rarity of this tumor.

## Conclusion

Our data provide further insight into the protein expression patterns of endolymphatic sac tumors. PSMA and SSTR2A, two well-established radiotracer targets, were not detected in the epithelial tumor cells in our cohort. However, PSMA expression was noted in the tumor neovasculature of all ELSTs, and SSTR2A expression in the neovasculature of the majority of ELSTs. These results corroborate noninvasive techniques such as ^68^Ga-DOTATATE-PET as a potentially useful screening tool in patients with suspected ELST. This observation awaits validation as a more sensitive screening and follow-up modality for ELSTs in prospective and multidisciplinary studies with larger cohorts.

## Data Availability

The data presented in this study are available on reasonable request from the corresponding author. The data are not publicly available due to ethical/privacy restrictions.
